# Concentrations of Phthalate Metabolites in Milk, Urine, Saliva, and Serum of Lactating North Carolina Women

**DOI:** 10.1289/ehp.11610

**Published:** 2008-08-22

**Authors:** Erin P. Hines, Antonia M. Calafat, Manori J. Silva, Pauline Mendola, Suzanne E. Fenton

**Affiliations:** 1 Reproductive Toxicology Division, Developmental Biology Branch, Office of Research and Development, National Health and Environmental Effects Research Laboratory, U.S. Environmental Protection Agency, Research Triangle Park, North Carolina, USA; 2 Division of Laboratory Science, National Center for Environmental Health, Centers for Disease Control and Prevention, Atlanta, Georgia, USA; 3 Infant, Child, and Women’s Health Statistics, U.S. Department of Health and Human Services, Centers for Disease Control and Prevention, National Center for Health Statistics, Hyattsville, Maryland, USA

**Keywords:** biomonitoring, breast milk, lactation, MAMA study, phthalates, saliva, serum, urine

## Abstract

**Background:**

Phthalates are ubiquitous in the environment, but concentrations in multiple media from breast-feeding U.S. women have not been evaluated.

**Objectives:**

The objective of this study was to accurately measure and compare the concentrations of oxidative monoester phthalate metabolites in milk and surrogate fluids (serum, saliva, and urine) of 33 lactating North Carolina women.

**Methods:**

We analyzed serum, saliva, urine, and milk for the oxidative phthalate metabolites mono(3-carboxypropyl) phthalate, mono(2-ethyl-5-carboxypentyl) phthalate (MECPP), mono(2-ethyl-5-hydroxyhexyl) phthalate, and mono(2-ethyl-5-oxohexyl) phthalate using isotope-dilution high-performance liquid chromatography tandem mass spectroscopy. Because only urine lacks esterases, we analyzed it for the hydrolytic phthalate monoesters.

**Results:**

We detected phthalate metabolites in few milk (< 10%) and saliva samples. MECPP was detected in > 80% of serum samples, but other metabolites were less common (3–22%). Seven of the 10 urinary metabolites were detectable in ≥ 85% of samples. Monoethyl phthalate had the highest mean concentration in urine. Metabolite concentrations differed by body fluid (urine > serum > milk and saliva). Questionnaire data suggest that frequent nail polish use, immunoglobulin A, and fasting serum glucose and triglyceride levels were increased among women with higher concentrations of urinary and/or serum phthalate metabolites; motor vehicle age was inversely correlated with certain urinary phthalate concentrations.

**Conclusions:**

Our data suggest that phthalate metabolites are most frequently detected in urine of lactating women and are less often detected in serum, milk, or saliva. Urinary phthalate concentrations reflect maternal exposure and do not represent the concentrations of oxidative metabolites in other body fluids, especially milk.

Phthalates, the diesters of phthalic acid, are ubiquitous in the environment, with annual global production at more than three million metric tons (Bizzari et al. 2000). Since their introduction in the 1930s, phthalates have been used as plasticizers and in cosmetics, food containers, medicine coatings, lubricants, adhesives, ink, medical devices, and tubing (Hauser and Calafat 2005). Certain phthalates have been shown to be endocrine disruptors in laboratory animals. The toxicity of phthalates in animals is related to the structure of the phthalate, the dose administered, and the animal’s age at exposure (Foster 2006; Foster et al. 2001). Rat testicular toxicity shows differential sensitivity based on the length of the phthalate alkyl side chain (Foster et al. 1980). Many of the effects in animals are seen at high, non-environmentally relevant doses (Parks et al. 2000). Young male rats exposed *in utero* or pubertally are more sensitive to the effects of phthalates than are adult animals (Foster 2006). Knowledge of the exposure of breast-feeding populations to phthalates is limited, and the distribution of phthalates in various bodily fluids during lactation is of interest for childhood nutrition and for exposure and health risk assessment.

Biomonitoring studies have shown phthalate exposure is widespread in humans (Duty et al. 2005; Silva et al. 2004). Phthalate exposure occurs via dermal contact, intravenous injection, inhalation, or ingestion. After exposure, phthalates are metabolized and excreted with an elimination half-life of 8–10 hr in adults (Bruns-Weller and Pfordt 1999). The half-life of phthalates in children or lactating women is unknown. All phthalates are first metabolized to their hydrolytic monoesters, and some phthalates can be further metabolized to their oxidative metabolites. The tendency to form oxidative metabolites increases as the molecular weight of the phthalate increases. Traditionally, the hydrolytic monoesters have been measured because they are considered to be biologically active. However, the exclusive use of the hydrolytic monoester metabolites underrepresents exposure to high-molecular-weight phthalates (Högberg et al. 2008; Silva et al. 2005b).

Few studies have evaluated phthalate concentrations in pregnant and lactating women. Monoethyl phthalate (MEP), monobutyl phthalate (MBP), monobenzyl phthalate (MBzP), and mono(2-ethylhexyl) phthalate (MEHP) have been detected in urine specimens, and their diester parent compounds in house dust samples, from pregnant women living in New York City (Adibi et al. 2003, 2008). Breast milk has been reported to contain phthalate metabolite monoesters in samples from Denmark/Finland (Main et al. 2006), Sweden (Högberg et al. 2008), and Italy (Latini et al. 2003). Calafat et al. (2004), in a method development study, followed the monoester and oxidative metabolites of three pooled U.S. human milk samples and found that most of the oxidative metabolites were at or below the limit of detection (LOD). The objectives of the present study were to accurately measure and compare the concentrations of oxidative monoester phthalate metabolites in milk and surrogate fluids (serum, saliva, and urine) of 33 lactating North Carolina (NC) women. We explored the interrelationship of phthalate metabolites detected in urine and serum, as well as potential associations with questionnaire exposure measures.

## Materials and Methods

### Use of human subjects

The U.S. Environmental Protection Agency (U.S. EPA) conducted the Methods Advancement for Milk Analysis (MAMA) study to evaluate the concentrations of endogenous and environmental components in human milk and to compare these concentrations to those in surrogate media including serum, saliva, and urine. We designed the MAMA study as a smaller methods development pilot for the longitudinal National Children’s Study that will follow 100,000 children from preconception to age 21 (Landrigan et al. 2006; National Children’s Study 2008). The MAMA study monitored 33 NC women over two time periods during lactation (2–7 weeks postpartum and 3–4 months postpartum). The participation of human subjects in the MAMA study was approved by the Institutional Review Boards (IRB) of the University of North Carolina at Chapel Hill School of Medicine (IRB no. 03-EPA-207) and the Centers for Disease Control and Prevention (CDC; IRB no. 3961). We briefed each woman on the study goals, risks, and inclusion/exclusion criteria and participated in informed consent (verbally and written) before completion of a comprehensive questionnaire, which did not include questions pertaining to the offspring of MAMA participants.

The women were recruited by an EPA contractor (Westat Inc., Chapel Hill, NC) via newspaper advertisements, university e-mail publications, and fliers distributed to clinicians specializing in women’s health or pediatrics. The women participated in the study at the EPA’s Human Studies Facility clinic (Chapel Hill, NC) between December 2004 and July 2005.

### Questionnaire

We administered a questionnaire about maternal exposure, occupation, residence, diet, and lifestyle to participants at the first clinic visit only. We designed the questions to address potential routes of exposure to multiple environmental chemicals (phthalates, phenols, perfluoroalkyl compounds, persistent organic pollutants, metals, and brominated flame retardants). As a pilot study, the MAMA questionnaire asked discrete questions (not open-ended) based on common exposures likely to be relevant to the general population, but did not independently validate the questions. We also compared biologics (serum and milk hormones, cytokines, glucose, triglycerides, and immunoglobulins), as previously reported (Hines et al. 2007), with questionnaire data and phthalate metabolite concentrations, paying special attention to end points that have been reported to be pertinent to phthalate exposure.

### Study design and sample collection

We recruited healthy, English-speaking women between 18 and 38 years of age who were breast-feeding their first, second, or third child. We did not require mothers to exclusively breast-feed. We asked them to fast for 1.5 hr before donating milk, saliva, urine, and serum at two established collection periods: 2–7 weeks and 3–4 months postpartum (visit 1 milk samples, *n* = 18; visit 2 milk samples, *n* = 20; visit 1 other fluids, *n* = 33; visit 2 other fluids, *n* = 30). We typically collected the urine specimen first, followed by serum, milk, and saliva. We recorded the sampling details, including time of day (between 0900 hr and 1400 hr) and the amount of bodily fluid collected, in the collection log. Milk (90 mL, or ~3 ounces) was expressed in the EPA clinic using a commercially available electric breast pump (Medela, McHenry, IL). All containers used in the collection and storage of the samples in this study were known to be phthalate-free based on earlier analyses by the CDC (data not shown). Before the MAMA study, we performed a leaching study in our lab to determine if any additional phthalates could be contributed to a milk sample collected by the breast pump chosen for this study (Hines EP, et al., unpublished data). This leaching study showed no significant difference in phthalate metabolite concentrations after passing through a breast pump. Milk was pumped into di(2-ethylhexyl) phthalate (DEHP)-free polypropylene bottles, divided into 3 mL aliquots in polypropylene tubes, and treated with 1 M phosphoric acid (125 μL/mL milk) to neutralize esterases. The women also donated about 20 mL of blood, which was collected into nonheparinized glass Vacutainer (Becton Dickinson, Franklin Lakes, NJ) tubes by an EPA nurse via venipuncture of the median cubital vein. After 1 hr at room temperature to allow for clotting, we spun blood samples at 3,000 rpm for 15 min at room temperature and collected the serum. If fibrin clotting of the serum layer occurred after the initial centrifugation, we ruptured the clots and centrifuged samples a second time. We treated serum samples with phosphoric acid as described above for milk. Saliva was collected in six polypropylene salivettes (Sarstedt AG, Nümbrecht, Germany) as described previously (Ferrari et al. 2008), and 3 mL of saliva was collected and transferred to a polypropylene cryovial and treated with phosphoric acid as described above. Similarly, we collected urine into polypropylene collection cups without further treatment and aliquoted (3 mL) into polypropylene cryovials. We stored all samples at −20°C and shipped them on dry ice to the CDC’s Division of Laboratory Sciences, National Center for Environmental Health (Atlanta, GA), for analysis. At the CDC, all samples were stored at or below −20°C until analyzed.

### Methods

We performed the preparation of standard solutions, quality control (QC) verification, sample preparation, and instrumental analyses as previously described (Calafat et al. 2004) with slight modification of the sample preparation as described herein. Sample analyses involved enzymatic deconjugation of the glucuronidated phthalates, automated solid-phase extraction, and separation using isotope-dilution high-performance liquid chromatography followed by tandem mass spectrometry (milk, Calafat et al. 2004; urine, Kato et al. 2004; serum and saliva, Silva et al. 2005a, 2005b). This high-throughput approach allows for simultaneous detection of at least 10 phthalate metabolites. We used internal standards (isotope-labeled and conjugated) to increase measurement precision and accuracy. We ran QC and reagent blank samples with unknown samples to monitor method performance.

We measured phthalate hydrolytic monoesters and oxidative metabolites in urine. Breast milk, serum, and saliva hydrolytic monoesters, were measured and sometimes detected but are not reported because environmental contamination of diesters and nearly concomitant generation of monoesters from these diesters can lead to an inflated representation of the milk (or surrogate fluid) monoester phthalate pool (Calafat et al. 2004). Environmental contamination can easily occur because serum is collected via Vacutainer and requires time to clot before multiple centrifugations occur, after which acid is added to samples. Similarly, milk collection is a multiple-step collection process with the possibility for environmental contamination during that time. We report the concentrations of oxidative metabolites for milk, serum, saliva, and urine. Milk, saliva, and serum lower limits of quantification (LOQs) were 1.07 μg/L [mono(3-carboxypropyl) phthalate (MCPP)], 0.80 μg/L [mono(2-ethyl-5-carboxypentyl) phthalate (MECPP)], 1.07 μg/L [mono(2-ethyl-5-hydroxyhexyl) phthalate (MEHHP)], and 0.80 μg/L [mono(2-ethyl-5-oxohexyl) phthalate (MEOHP)]. In urine, the LODs were 0.45 μg/L (MBP), 0.40 μg/L (MBzP), 0.32 μg/L (MCPP), 0.40 μg/L (MECPP), 0.11 μg/L (MEHHP), 0.25 μg/L (MEHP), 0.16 μg/L (MEOHP), 0.26 μg/L (MEP), 1.00 μg/L [monomethyl phthalate (MMP)], and 0.90 μg/L [monoisobutyl phthalate (MiBP)]. Breast milk phthalate metabolite concentrations can fall below LOD due to concentration factors. We report concentrations as micrograms per liter in serum, milk, and saliva, and in urine both as micrograms per liter and as micrograms per gram creatinine after creatinine adjustment to correct for urine dilution. We analyzed urine samples for creatinine using a Beckman Synchron AS/ASTRA clinical analyzer (Beckman Instruments, Inc., Brea, CA) at the CDC. A previous study (Adibi et al. 2008) in pregnant women suggested that specific gravity was a better indicator of urine dilution than is creatinine. Because this was unknown at the time of our data analysis, we measured creatinine. MEP, the smallest of the metabolites reported here, may have a different excretion pattern than other phthalates, and thus creatinine adjustment may alter MEP’s values differently than those of other phthalate metabolites (Boeniger et al. 1993; Duty et al. 2003). Thus, most comparisons in this article focus on urine concentrations of phthalates independent of creatinine adjustment.

### Statistics

We did not compare phthalate metabolites across matrices when we detected phthalates in < 50% of samples (Baccarelli et al. 2005). We assessed Spearman correlations to determine relationships within and between phthalate metabolites, comparing visit 1 and visit 2 unadjusted and creatinine-adjusted urinary concentrations. We treated values lower than the LOD as missing for all analyses. We used rank order correlations to account for the nonparametric distribution of the phthalate metabolites. Where serum phthalate metabolite concentrations for visits 1 and 2 were detectable, we compared serum and urine concentrations using Spearman correlations. For categorical questionnaire measures, we used one-way analysis of variance to test for differences in mean phthalate metabolite concentrations, using both the original metabolite levels and repeated after transforming the data to a log-normal distribution with a mean of 0 and standard deviation of 1. The association between continuous questionnaire measures and phthalate metabolite concentrations was assessed with Spearman correlations. We used paired *t*-tests to compare the total urinary metabolite concentrations and a subset of DEHP metabolite concentrations from visit 1 to visit 2. No adjustments were made for multiple comparisons. We conducted all analyses using SAS Enterprise Guide 4.1 (SAS Institute Inc. 2006).

## Results

### Phthalate metabolites detected in MAMA samples

Table 1 lists the percentages of detectable phthalate metabolite concentrations. Of the total milk samples, 3%, 8%, 5%, and 2% contained detectable concentrations of MCPP, MECPP, MEHHP, and MEOHP, respectively. Of the total saliva samples, 2%, 2%, 0%, and 0% contained detectable concentrations of MCPP, MECPP, MEHHP, and MEOHP, respectively. We detected urinary phthalate metabolites in > 85% of the urine samples, with the exception of MMP, MEHP, and MiBP (Table 1). We detected MCPP, MECPP, MEHHP, and MEOHP in 3%, 87%, 17%, and 14% of total serum samples, respectively. Because we found MECPP in > 50% of serum samples, we compared it across matrices (serum vs. urine).

### Concentrations of phthalate metabolites in human milk

We detected MCPP, MECPP, MEHHP, and MEOHP in very few breast milk samples (in low parts per billion concentrations). When we considered the data from both collection times, we detected two analytes in 1 of 38 samples from 65 analytic runs (MCPP, 0.2 μg/L; MEOHP, 0.3 μg/L). We detected MECPP in 5 of 39 samples from 65 analytic runs (0.1, 0.1, 0.1, 0.3, and 0.4 μg/L), and MEHHP in 3 of 39 samples from 65 analytic runs (0.2, 0.3, and 0.3 μg/L). There appeared to be insignificant concentrations of phthalate oxidative metabolites in breast milk collected in this study.

### Concentrations of phthalate metabolites in serum

**Figure 1:**
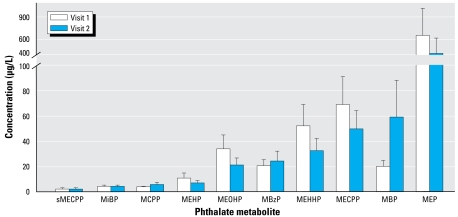
Mean ± SE phthalate metabolite levels (μg/L) from visit 1 and visit 2 serum (sMECPP) and urine (all other metabolites).

We measured the oxidative phthalate monoesters MCPP, MECPP, MEHHP, and MEOHP in human serum. MECPP was detected in the highest amounts with a mean of 2.0 μg/L (visit 1) and 2.3 μg/L (visit 2) (range, < LOD to 13.7 μg/L). We detected the other metabolites less frequently, and their means were close to their respective LOQs. Figure 1 shows serum MECPP concentrations for visits 1 and 2.

### Concentrations of phthalate metabolites in saliva

We detected only two phthalate metabolites (2.2 μg/L MCPP and 2.3 μg/L MECPP) in a single saliva sample from one woman on one visit. All other phthalate concentrations were undetectable. These data suggest limited transfer of oxidative phthalate metabolites into saliva in a fasting breast-feeding mother.

### Concentrations of phthalate metabolites in urine

Table 2 shows median and selected percentiles of phthalate metabolite concentrations in urine. Urine, unlike the other biologic media analyzed in this study, does not contain esterases, which can break down parent diester phthalates to hydrolytic monoester metabolites. Therefore, we report all monoester metabolite concentrations in urine because contamination contribution to the monoester metabolite pool is unlikely. Four of these metabolites, MEHP, MECPP, MEHHP, and MEOHP, are derived from the same parent compound, DEHP (as detailed in Koch et al. 2005). MEP concentrations, on average, were 10-fold higher than the next highest phthalate metabolite (Figure 1).

### Total urinary phthalate load

**Figure 2:**
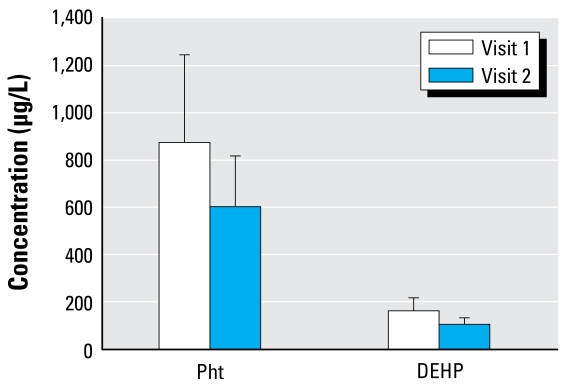
Urinary concentrations (mean ± SE) of total phthalate metabolites (Pht) and DEHP phthalate metabolites (μg/L, unadjusted), by visit.

Figure 2 reports total phthalate metabolite concentrations and the total concentration of DEHP metabolites in urine, by visit. We measured multiple DEHP metabolites in this study, including MEHP, its monoester metabolite, and its oxidative metabolites (MEHHP, MEOHP, and MECPP). We found no significant change in both total and DEHP-derived metabolites between visits (by two-tailed *t*-test, *p* < 0.05) because urinary metabolite concentrations tended to vary greatly within each subject across the visits.

### Correlations

#### Between- and within-visit correlations—individual urinary metabolites

As anticipated, all of the unadjusted urinary metabolite concentrations were significantly correlated with the creatinine-adjusted levels from the same visit (Spearman’s *R* ranged from 0.50 to 0.78; data not shown). MMP was not correlated between visits, which may be confounded by MMP detected in less than 20% of the samples. Visit 1 concentrations of the other nine urinary metabolites were significantly correlated with their measurements at visit 2 when examining the unadjusted values (Table 3), but only MBzP and MEP were significantly correlated between visits 1 and 2 for creatinine-adjusted concentrations.

#### Comparing serum with urinary metabolites

Only MECPP had sufficient detection frequency in serum to allow for comparison between urine and serum concentrations (Table 4). Serum and urine MECPP concentrations were correlated within visits but not between visits (Spearman *R* = −0.22, *p* = 0.27, *n* = 28). Table 5 shows the relation of serum MECPP concentrations to those of other DEHP metabolites in urine. At visit 1, MECPP in serum was correlated with urinary MEHHP (unadjusted only), MEHP (creatinine-adjusted only), and MEOHP (both unadjusted and adjusted concentrations). At visit 2, MECPP in serum was correlated with MEHHP in urine (unadjusted and adjusted for creatinine) and with MEOHP (unadjusted only). Serum MECPP was not correlated with urinary MEHP at visit 2. No serum measure was correlated with any urine measure from the other visit.

#### Correlations between urinary metabolites of DEHP

Within visits, correlations among the DEHP urine metabolites (the hydrolytic monoester MEHP and three of its oxidative products, MECPP, MEHHP, and MEOHP) were strongest for MECPP and MEHHP (Spearman’s *R* ranged from 0.96 to 0.61; data not shown) and MEOHP (Spearman’s *R* ranged from 0.97 to 0.61; data not shown). Unadjusted MEHP concentrations were correlated with both unadjusted MECPP (*R* = 0.62, *p* < 0.001) and adjusted MECPP concentrations (*R* = 0.48, *p* < 0.01) at visit 1 but not at visit 2. MEOHP and MEHHP were also generally well correlated within visits (*R* ranged from 0.98 to 0.62; data not shown). Overall, the DEHP metabo lites correlated well with each other, with the oxidative metabolites having stronger correlation among themselves than with the monoester MEHP.

#### Correlations of phthalate metabolites with questionnaire data

We administered the questionnaire only at visit 1, so we compared questionnaire data with visit 1 phthalate metabolite concentrations. Because previous work has shown that creatinine is not the best way to adjust urinary phthalate levels (Adibi et al. 2008) and because creatinine-adjusted and nonadjusted phthalate values correlate within visits, we used nonadjusted urinary concentrations for the remainder of the data analysis. Most of the questionnaire variables were not significantly related to metabolite concentrations, including the number of prior births, prior breast-feeding, use of prescription or over-the-counter medication, new furniture, blinds, and use of glues or solvents, hair products, and paint. Using the untransformed metabolite data, serum MECPP was significantly different by income category, with the highest metabolite concentrations in the middle income group ($50,000–70,000), but after log-normal transformation, this was no longer statistically significant (*p* = 0.15). We observed no relation for income with any of the urinary metabolite concentrations. Untransformed serum MECPP concentrations differed significantly by race (*F* = 4.90, *p* = 0.04, df = 1), with higher mean levels among nonwhites (mean ± SD = 5.03 ± 5.64) compared with white mothers (1.84 ± 2.09), but again, this was not significant after log transformation of the raw data (*p* = 0.13). Mean levels of urinary MEP increased significantly according to self-reported nail polish use in the untransformed data, with a mean level of 2873.8 (SD = 1826.0) for women who “often” use nail polish compared with 50.5 (SD = 11.6) for women who “never” use it. After log transformation, we observed additional associations between nail polish use (any vs. none) and MBP (*p* < 0.001), MBzP (*p* < 0.01), MECPP (*p* = 0.01), MEHHP (*p* < 0.01), MEOHP (*p* < 0.01), and the sum of DEHP metabolites (*p* < 0.01). Interestingly, MECPP, MEHHP, MEOHP, and MiBP concentrations were all inversely correlated with the age of the primary car driven by the participants (Table 6); women who drive newer cars appear to have higher urinary concentrations of these phthalate metabolites.

#### Immune-related correlations

Serum immunoglobulin A (IgA) and urinary MBP at visit 1 were correlated (rho = 0.36, *p* = 0.04, *n* = 32). No other phthalate metabolites correlated with serum IgA. Serum IgM, IgE, and IgG showed no significant correlations with phthalate metabolite concentrations.

#### Glucose-related correlations

Multiple glucose-related end points showed correlations with phthalate exposure. Although only two mothers had reported gestational diabetes, both their serum and urine concentrations of MECPP were more than three times the mean of mothers without reported gestational diabetes (serum, 6.8 ng/mL vs. 1.9 ng/mL; urine, 212.9 ng/mL vs. 59.8 ng/mL). Several of the urinary DEHP metabolites (MECPP, MEHP, MEHHP, and MEOHP) in visit 1 samples correlated with visit 1 serum glucose (Table 7); only three of the MAMA participants had glucose levels outside the normal range (≥100 mg/dL at fasting), and only one of these three reported gestational diabetes.

#### Triglyceride-related correlations

Multiple phthalate metabolites showed significant positive correlations with serum triglycerides. Visit 1 urinary MEHP, MECPP, MEHHP, and MEOHP concentrations all correlated with visit 1 serum triglyceride concentrations (Table 8). None of the other phthalate metabolites correlated with serum triglycerides at visit 1. Eight of the 33 women had serum triglyceride levels defined as high for healthy nonlactating adults; normal levels are not available for lactating women.

#### Nonsignificant findings

Comparing phthalate MAMA metabolite concentrations (urine and serum) with a biologics panel (Hines et al. 2007) from serum collected at visit 1, which included IgG, IgE, IgM, prolactin, estradiol, tumor necrosis factor-alpha, and interleukin-6, yielded nonsignificant findings.

## Discussion

Lactational exposure of U.S. infants to phthalates has largely been overlooked. The MAMA study is the first to evaluate phthalate concentrations in multiple body fluids from a convenience sample of lactating women in the United States at two time points, focusing on oxidative phthalate metabolites in serum, milk, and saliva and both oxidative and hydrolytic monoester metabolites in urine. We detected phthalate oxidative metabolites (MCPP, MECPP, MEHHP, and MEOHP) in very few milk samples, and those that we detected were near the LOQ. Because of the methods development nature of this study, we analyzed breast milk samples from 55% of mothers at visit 1 and 67% at visit 2, in contrast to other fluids that we analyzed for nearly every woman. Nevertheless, very few milk samples had any detectable phthalate metabolites, making comparisons with other biologic fluids impossible. Given these findings, milk is unlikely to contain oxidative phthalate metabolites, and investigators interested in exposure assessment in lactating women should focus on collecting urine. Further, we detected only two metabolites in saliva from one woman on one visit; all other phthalates were undetectable in saliva. This suggests that there is little transfer of phthalates to saliva of fasting lactating women.

Several phthalate assessment studies have been conducted previously, including a Canadian study on milk diesters (Zhu et al. 2006), a Japanese study on breast milk hydrolytic monoesters (Takatori et al. 2007), and an Italian study on diesters in milk (Latini et al. 2003). A previous study on phthalate metabolites in multiple media (milk, urine, and serum) from Swedish women abstaining from skin care product use collected milk and 1 week later collected urine and serum (Högberg et al. 2008). In this study, we collected all body fluids at one time in women with no personal care product use limitations. Our rates of detection are in agreement with previous studies, including Högberg et al.’s (2008) findings, except that in this study we detected MiBP less frequently than did the Adibi et al. (2008) study. In Main et al.’s (2006) study of Danish and Finnish breast milk, samples used for hydrolytic monoester phthalate metabolite analysis were pooled from multiple small hindmilk collections 1–3 months postpartum.

Urinary phthalate concentrations in the present study were comparable with those in studies of pregnant women in the United States and abroad (Adibi et al. 2003; Swan et al. 2005), with MEP concentrations in these studies 10-fold greater than other monoester metabolites. Phthalate exposure comparisons were made using non-creatinine-adjusted values based on earlier work by Adibi et al. (2008). Mean urinary MEP and MEHP concentrations detected in this study were similar (2-fold higher) to those in females or people 20–39 years of age as reported for the 1999–2000 National Health and Nutrition Examination Survey (NHANES) (Silva et al. 2004). Urinary MBP and MBzP concentrations in this study were also similar to the NHANES values.

Several human studies have reported correlations between possible health effects and low concentrations of phthalate metabolites. Duty et al. (2003) found a dose–response relationship between urinary MBP or MBzP concentrations and sperm motility and/or sperm concentration (indicators of sperm quality). Swan et al. (2005) found that in human male infants, anogenital distance, previously found to be a sensitive marker of antiandrogen activity in rodents, was inversely related to urinary concentrations of some phthalates during pregnancy. Wolff et al. (2008) found that low-molecular-weight urinary phthalate metabolites (as measured in the third trimester of pregnancy) were positively correlated with gestational age and head circumference of newborn children. Recent analyses of NHANES data revealed positive correlations between urinary phthalate metabolite concentrations and glucose and obesity-related end points (Hatch et al. 2008; Stahlhut et al. 2007).

In this study, MECPP was the most frequently detected metabolite in serum, with a mean value of 2 μg/L; other oxidative phthalate metabolites are not above LOQ in most serum samples (78–97% not detected). Other human studies have reported serum phthalate monoester concentrations in Chinese workers with occupational phthalate exposure (Pan et al. 2006).

Our results demonstrate that phthalate metabolites are unlikely to be detectable in milk and saliva. However, serum MECPP and urinary phthalate metabolites (except MMP) are informative end points for evaluation of the exposure to lactating women, but this information does not translate into lactational exposure to the infant. Those phthalate metabolites that were detectable showed several potentially important relationships with questionnaire or biologic end points.

Biomonitoring of phthalates in human populations has revealed differences among racial/ethnic groups (Silva et al. 2004; Wolff et al. 2007). In this study, whites had lower mean MECPP serum concentrations than did nonwhites (2.1 and 5.0 μg/L, respectively) at visit 1, but because 91% of the participants were white, this result has low statistical power. In NHANES 1999–2000, urinary concentrations of MEP were significantly higher in non-Hispanic blacks than in other race/ethnicity groups, and in non-Hispanic black children than in non-Hispanic white and Mexican-American children (Silva et al. 2004). In another study, white adolescent girls had lower concentrations of MEHP and MEP but higher concentrations of MCPP than did other race/ethnicity groups (Asian, black, and Hispanic) (Wolff et al. 2007). Consensus in racial differences across studies may be due to differences between ethnic groups in phthalate-metabolizing enzymes, specifically polymorphisms in UDP-glucuronyltransferase genes (de Wildt et al. 1999; Dirven et al. 1993), but too few nonwhites participated in the present study for us to explore this interesting possibility.

Self-reported nail polish use showed a strong relation to untransformed urinary MEP concentrations, and several metabolites were associated with nail polish use after log transformation (MBP, MBzP, MECPP, MEHHP, MEOHP, and the sum of DEHP metabolites). Personal care products, including nail polish, have been reported to contain diethyl phthalate (DEP), the parent compound of MEP (Kamrin and Mayor 1991). It is also possible that this metabolite reflects that women who used nail polish also used perfume and other personal care products that may contain DEP (Houlihan et al. 2002; Hubinger and Harvery 2006; Koo and Lee 2004). Similar to the present study, Duty et al. (2005) found that personal care product use predicted urinary MEP concentrations in a cohort of men and that MEP increased for each additional type of product used. A recent pediatric study found increased urinary phthalate metabolite levels in infants after topical application of baby products, including lotions, soaps, and powders (Sathyanarayana et al. 2008). We also found an inverse correlation between primary motor vehicle age and urinary phthalate metabolite concentrations, including MiBP and the oxidative metabolites of DEHP. Phthalates have been previously reported to be found in indoor and personal air (Adibi et al. 2008; Rudel et al. 2003), which could explain an inhalation route of exposure.

We and others have found correlations between phthalate metabolites and serum glucose concentrations. In two recent studies, analyses of NHANES urinary phthalate metabolites found correlations between certain phthalate monoester or oxidative metabolites and abdominal obesity or homeostasis model assessment index (HOMA), a biomarker for insulin resistance (Hatch et al. 2008; Stahlhut et al. 2007). We found increased MECPP concentrations in serum for mothers with self-reported maternal gestational diabetes (*n* = 2) compared with the other mothers, and serum glucose levels were correlated with DEHP metabolite concentrations (visit 1: serum MECPP and urinary MECPP, MEHHP, and MEOHP). Hatch et al. (2008), Stahlhut et al. (2007), and our data all showed correlations of glucose-related outcomes with DEHP metabolites, including MEHHP and MEOHP. Although we did not measure the health outcomes that were addressed in those prior studies, similar concentrations of the phthalate metabolites were observed in all three studies, suggesting that environmentally relevant exposure levels may translate into health-related outcomes. Future research in this area needs to address mechanisms for these health outcomes.

In the present study, serum triglycerides and DEHP metabolites were also correlated. Studies in mice have reported that subchronic or chronic phthalate exposure leads to increased serum and/or liver tri glycerides (Mapuskar et al. 2007; Pereira et al. 2006). Acute exposures to DEHP and/or di(*n*-hexyl)phthalate in rodents is associated with decreased triglyceride levels (Howarth et al. 2001). A rat study using an intermediate dosing regimen of 21 days found a decrease in total triglycerides that was mediated via lipase induction (Mocchiutti and Bernal 1997). Therefore, it appears that dose and duration of phthalate exposure in the rodent models differentially regulated serum triglyceride levels, but these associations have not been explored in humans.

In this study, we also found a correlation between serum IgA concentration (Hines et al. 2007) and urinary MBP concentration. The role of phthalates as immunomodulators is just beginning to be explored. Genetically susceptible mice developed autoimmunity after DEHP exposure (Lim and Ghosh 2005). The association of phthalates with epidemologic respiratory immunity changes is highly debated (Doelman et al. 1990; Jaakkola et al. 1999; Mendell 2007; Oie et al. 1997). Studies in other labs have shown up-regulation of the immunosuppressive FK506 binding protein family of genes, specifically *FKBP-1* and *FKBP-13*, after diisononyl phthalate exposure in rodents (Valles et al. 2003).

Finally, earlier work has shown that human urinary phthalate concentrations vary over the course of a day (Janjua et al. 2008). Women in this study visited the clinic between 0900 hr and 1400 hr, and this window could lead to some temporal variability. However, there were no significant differences in total phthalate metabolite load or DEHP metabolite load of our participants across visits to the clinic. In fact, we found significant correlations of 9 of the 10 phthalate urinary metabolites with themselves between visits when reported without creatinine adjustment. Taking the findings of all of these studies into consideration, the use of a single sample collection to define continuing phthalate exposure may be adequate to reflect average phthalate exposure.

## Conclusions

In lactating NC women, we found detectable concentrations of oxidative phthalate metabolites in very few breast milk samples. Further, these same women had detectable concentrations of oxidative and hydrolytic monoester phthalate metabolites in nearly all of their urine samples, detectable concentrations of MECPP in > 80% of their serum samples, and almost no detectable concentrations of phthalate metabolites in their saliva. Thus, surrogate fluid measures (i.e., urine, serum, saliva) are not a good proxy measure for milk levels. In particular, high urinary metabolite levels do not predict detectable phthalates in human milk.

We examined the relationship of urinary metabolites, serum MECPP, questionnaire, and other biologic measures. The concentrations of DEHP metabolites showed significant correlation, pointing to a common parent compound for exposure. Interestingly, metabolism-related end points (glucose and triglycerides) were significantly associated with phthalate metabolite concentrations. Potential sources of phthalate exposure such as nail polish use and newer cars were significantly associated with increased concentrations of some phthalate metabolites. The total and DEHP-derived phthalate loads for study participants were not significantly different over time. Further, it appears that a single sampling may accurately represent average environmental phthalate exposure to the mother.

Breast milk is a superior source of infant nutrition with social, economic, and nutritional benefits. The infrequent detection of measurable oxidative phthalate metabolites in milk, even in women with multiple metabolites in their urine, supports continued efforts to promote breast-feeding worldwide.

## Figures and Tables

**Table 1:** Various phthalate metabolites detected (% > LOD) in samples from lactating NC women: U.S. EPA MAMA study, 2004–2005.

	Phthalate metabolite										
Fluid	MCPP	MECPP	MEHHP	MEOHP	MBP	MBzP	MEHP	MEP	MMP	MiBP	No.^a^
Milk											
Visit 1	6	8	8	4	NA	NA	NA	NA	NA	NA	18
Visit 2	0	8	3	0	NA	NA	NA	NA	NA	NA	20
Total	3	8	5	2	NA	NA	NA	NA	NA	NA	38
Serum											
Visit 1	3	84	22	19	NA	NA	NA	NA	NA	NA	32
Visit 2	3	93	13	13	NA	NA	NA	NA	NA	NA	30
Total	3	87	17	14	NA	NA	NA	NA	NA	NA	62
Saliva											
Visit 1	0	0	0	0	NA	NA	NA	NA	NA	NA	32
Visit 2	3	3	0	0	NA	NA	NA	NA	NA	NA	30
Total	2	2	0	0	NA	NA	NA	NA	NA	NA	62
Urine											
Visit 1	100	100	100	100	97	100	91	100	18	97	33
Visit 2	93	100	93	93	80	100	50	100	7	67	30
Total	97	100	97	97	89	100	71	100	13	82	63

NA, not analyzed.

a Number of samples measured for each end point.

**Table 2:** Median and selected percentiles of phthalate metabolite concentrations in urine from lactating NC women: U.S. EPA MAMA Study, 2004–2005.

Phthalate metabolite	Visit	10th percentile	25th percentile	Median	75th percentile	90th percentile	95th percentile	No.^a^
MBP	1	1.3 (9.7)	6.8 (13.4)	14 (18.3)	21.8 (28.1)	38.9 (34.9)	53.5 (40.8)	33
	2	< LOD (4.3)	2.1 (11.0)	6.6 (18.2)	9.5 (48.0)	94.2 (71.3)	155.9 (133.0)	30
MBzP	1	1.6 (6.3)	4.2 (9.2)	9.6 (14.4)	27.7 (32.1)	52.6 (49.7)	75.6 (70.2)	33
	2	1.0 (5.1)	3.8 (7.6)	11.0 (13.4)	22.5 (27.2)	53.2 (56.4)	141.1 (62.6)	30
MCPP	1	0.6 (2.3)	1.5 (2.9)	3.2 (3.5)	5.4 (6.1)	7.6 (7.2)	11.4 (17.9)	33
	2	0.4 (1.3)	0.8 (2.7)	3.4 (3.7)	6.2 (7.1)	14.2 (9.0)	18.2 (10.2)	30
MECPP	1	4.2 (16.8)	12.8 (23.0)	27.3 (36.8)	51.9 (89.1)	218.3 (152.9)	364.4 (247.9)	33
	2	3.1 (12.7)	6.7 (18.3)	24.8 (32.5)	58.0 (64.4)	138.9 (102.5)	236.9 (134.8)	30
MEHHP	1	3.6 (10.5)	9.8 (14.4)	18.6 (24.5)	28.8 (43.9)	139.4 (153.9)	336.2 (183.0)	33
	2	1.4 (4.5)	4.2 (10.5)	10.9 (17.0)	32.6 (43.6)	107.8 (74.0)	181.9 (80.7)	30
MEHP	1	1.5 (1.9)	2.1 (3.4)	3.0 (7.6)	6.2 (15.1)	18.1 (30.3)	86.2 (46.9)	33
	2	< LOD (1.3)	< LOD (1.6)	< LOD (3.6)	4.3 (11.9)	13.4 (17.0)	21.1 (58.0)	30
MEOHP	1	2.6 (7.3)	6.5 (10.6)	12.0 (17.9)	19.3 (29.4)	77.1 (91.5)	224.2 (122.1)	33
	2	1.3 (4.3)	2.9 (8.6)	8.4 (13.5)	20.4 (32.5)	59.6 (43.9)	77.8 (45.2)	30
MEP	1	9.2 (28.7)	29.5 (48.5)	73.1 (145.0)	145.0 (218.5)	442.5 (535.1)	6891.8 (3155.6)	33
	2	13.2 (29.0)	23.2 (63.9)	74.3 (113.0)	275.2 (249.5)	996.1 (661.8)	1662.2 (2444.4)	30
MMP	1	< LOD (2.2)	< LOD (3.8)	< LOD (4.1)	< LOD (10.7)	2.6 (97.1)	4.5 (97.1)	33
	2	< LOD (3.5)	< LOD (3.5)	< LOD (4.9)	< LOD (6.4)	< LOD (6.4)	4.8 (6.4)	30
MiBP	1	0.7 (2.6)	1.5 (3.8)	3.8 (5.2)	6.6 (7.0)	10.0 (9.4)	12.1 (13.9)	33
	2	0.6 (1.0)	0.6 (1.9)	2.1 (3.4)	4.7 (7.6)	13.0 (10.5)	19.0 (12.2)	30

Abbreviations: U, unadjusted for creatinine (μg/L); U_A_, creatinine adjusted (μg/g). Values shown are U(U_A_).

a Numbers of samples measured for each end point.

**Table 3:** Spearman correlations for visit 1 versus visit 2 for urinary phthalate metabolites.

Correlation variable	MBP	MBzP	MCPP	MECPP	MEHHP	MEHP	MEOHP	MEP	MiBP
Unadjusted									
Rho	0.47	0.71	0.44	0.48	0.38	0.63	0.45	0.60	0.58
*p*-Value	0.02	< 0.0001	0.01	0.008	0.04	0.01	0.02	0.0005	0.0008
No.	24	30	30	30	30	15	28	30	30
Creatinine-adjusted									
Rho	0.24	0.54	0.31	0.29	0.24	0.48	0.20	0.50	0.33
*p*-Value	0.27	0.002	0.09	0.11	0.20	0.07	0.31	0.005	0.07
No.	24	30	30	30	30	15	28	30	30

**Table 4:** Spearman correlations between serum and unadjusted (U) and creatinine-adjusted (U_A_) urine MECPP levels for visits 1 and 2.

	Visit 1		Visit 2	
Serum	U	U_A_	U	U_A_
Visit 1	0.45	0.74	−0.12	0.08
*p*-Value	0.01	< 0.0001	0.53	0.70
No.	31	31	28	28
Visit 2	0.23	0.01	0.62	0.56
*p*-Value	0.21	0.97	0.0002	0.001
No.	30	30	3	30

**Table 5:** Spearman correlations between serum MECPP and other DEHP metabolite concentrations in urine.

	MEHHP		MEHP		MEOHP	
Sample	U	U_A_	U	U_A_	U	U_A_
Visit 1	0.43	−0.11	0.33	0.38	0.39	0.67
*p*-Value	0.02	0.57	0.08	0.04	0.03	< 0.0001
No.	31	28	28	28	31	31
Visit 2	0.50	0.43	0.31	0.18	0.42	0.35
*p*-Value	0.005	0.02	0.27	0.51	0.03	0.07
No.	30	30	15	28	28	28

Abbreviations: U, unadjusted; U_A_, creatinine adjusted.

**Table 6:** Significant correlations between car age and urinary phthalate metabolite concentrations at visit 1 (*n* = 30).

Variable	Spearman’s rho	*p*-Value
MECPP	−0.37	< 0.05
MEHHP	−0.39	0.03
MEOHP	−0.39	0.03
MiBP	−0.36	< 0.05

**Table 7:** Significant correlations between serum glucose concentrations and urinary phthalate metabolite concentrations at visit 1.

Variable	No.	Spearman’s rho	*p*-Value
MEHP	30	0.43	0.02
MECPP	33	0.35	< 0.05
MEHHP	33	0.37	0.03
MEOHP	33	0.38	0.03

**Table 8:** Significant correlations between serum triglycerides and urinary phthalate metabolites at visit 1.

Variable	No.	Spearman’s rho	*p*-Value
MEHP	30	0.48	< 0.01
MECPP	33	0.44	0.01
MEHHP	33	0.41	0.02
MEOHP	33	0.42	0.01
